# *Plasmodium malariae* in Haitian Refugees, Jamaica

**DOI:** 10.3201/eid1306.061227

**Published:** 2007-06

**Authors:** John F. Lindo, Jeanette Horner Bryce, Marion Bullock Ducasse, Christina Howitt, Donnett M. Barrett, Jacob Lorenzo Morales, Rosalynn Ord, Martina Burke, Peter L. Chiodini, Colin J. Sutherland

**Affiliations:** *Plasmodium malariae* in Haitian Refugees; * University of the West Indies, Kingston, Jamaica; † Ministry of Health, Kingston, Jamaica; ‡ London School of Hygiene and Tropical Medicine, London, UK; § University of La Laguna, Tenerife, Canary Islands, Spain; ¶ Hospital for Tropical Diseases, London, UK

**Keywords:** Plasmodium malariae, Plasmodium brasilianum, Haiti, refugees, Jamaica, dispatch

## Abstract

Since 1963, reported malaria transmission in Haiti has been restricted to *Plasmodium falciparum*. However, screening of Haitian refugees in Jamaica in 2004, by microscopic examination, identified *P. falciparum, P. vivax,* and *P. malariae.* PCR confirmed the *P. malariae* and *P. falciparum* but not *P. vivax* infections. DNA sequencing and rRNA gene sequences showed transmission of *P. malariae*. This report confirms that *P. malariae* is still being transmitted in Haiti.

Malaria remains an important disease in Latin America and the Caribbean, where 30.4% (264 million) of the 869 million persons live in areas where ecologic conditions have been propitious for the transmission of malaria (*1*). Endemic transmission of malaria in Jamaica was interrupted from 1960 through 1965, when the last cases (16 *Plasmodium malariae* and 1 *P. vivax*) were reported (*2*). The island remains at risk for reintroduction of malaria because the population is immunologically naive and the vector, *Anopheles albimanus,* is endemic there (*3*). One possible route of reintroduction is through infected persons from a malaria-endemic area such as Haiti. Transmission of endemic malaria in Haiti has been reported to be restricted to *P. falciparum;* the last cases of *P. vivax* were reported in 1983 and the last case of *P. malariae* in 1963 (*1*,*2*,*4*,*5*). *P. falciparum,* which was associated with Haitian immigrants, was the sole etiologic agent of reintroduced malaria, which caused recent an epidemic in Great Exuma Island, Bahamas (*6*).

## The Study

From February through April 2004, 429 Haitian refugees arrived by boat in Jamaica. Included in their health status screening was a microscopy examination for *P. falciparum* because malaria is well established in Haiti but is not endemic in Jamaica.

The refugees landed on Jamaica’s northeast shore, where they received chloroquine and primaquine and had blood was drawn for thick and thin blood film preparation. The smears were then transported over land to the National Public Health Laboratory in Kingston for microscopic examination.

Of the samples from the 429 refugees, a subset of 274 (which included posttreatment duplicates) was chosen by the Ministry of Health for inclusion in this aspect of the study. These samples included 30 *P. falciparum* isolates, 13 *P. vivax* isolates, and 1 isolate of *P. malariae* based on microscopy examination; 31 samples were identified only as *Plasmodium* sp. The lack of a definitive diagnosis may have been influenced by the quality of the smears made at first contact with the patients and the fact that some smears were taken after initial treatment and contained only dying or dead parasites.

Samples from 94 patients (including some negative by microscopy examination as well as posttreatment duplicates) were sent to the Heath Protection Agency, Malaria Reference Laboratory, London School of Hygiene and Tropical Medicine, for species confirmation by PCR. DNA was extracted from 105 filter-paper blood spots and tested for DNA from *P. falciparum*. *P. malaria,* or *P. vivax* by using either the single-round PCR protocol of Padley et al. (*7*) or the more sensitive nested protocol of Snounou et al. (*8*). Of 15 isolates that were positive for *P. malariae* DNA by either or both PCR procedures, 7 also harbored *P. falciparum* DNA. Only 1 of the PCR-confirmed *P. malariae* cases had been identified as such by microscopy. Of the remainder, 2 samples contained *P. vivax;* 1 contained *P. falciparum,* and 4 were free of malaria parasites. Of the 105 isolates tested, 57 were positive for *P. falciparum* DNA.

No cases of *P. vivax* were found with PCR. The absence of *P. vivax* was verified by species-specific real-time PCR, as described by de Monbrison et al. (*9*)

Four *P. malariae* isolates were selected for DNA sequencing, and the rRNA gene sequences were amplified by using a hybrid nested PCR approach. The first-round PCR products were produced by using the first stage of the nested PCR protocol; in the second round, the *P. malariae*–specific primers of Padley et al. (*7*), which lie wholly within the first-round PCR product, were used to amplify a final product of 425 bp. This was done because the much smaller nested PCR product produced by the protocol of Snounou et al. (*8*) (125 bp) was not suitable for direct sequencing. Products were purified by using the QIA Quick DNA extraction kit (QIAGEN, Crawley, UK), and directly sequenced on both strands by using the ABI BigDye sequencing kit (Applied Biosystems, Warrington, UK) according to the manufacturer’s instructions, except that the reaction mix was diluted 8-fold before use. The forward and reverse PCR primers were used to prime the sequencing reaction. Reaction products were fractionated on an ABI capillary sequencer (Applied Biosystems), and sequencing data were proofread with Chromas software (available from www.technylesium.com).

The full sequence obtained from both strands of 1 representative isolate was compared with 3 *P. malariae* rRNA gene sequences in GenBank, and to that of *P. brasilianum* that had been isolated from a monkey in French Guiana ([10]; GenBank accession no. AF130735) by using the program T-Coffee (http://ca.expasy.org/tools/#translate). The alignment produced is presented in the Figure. The Haitian sequence obtained matched very closely with all 4 *P. malariae* rRNA gene sequences in GenBank and with that of the indistinguishable simian parasite, *P. brasilianum* (*10*). Two single-nucleotide substitutions were observed in the sequence at positions 228 and 337, and each of these also occurred in at least 1 of the other 4 Haitian sequences we obtained (data not shown). Available sequence data for *P. malariae* are very scanty, and currently no well-established loci exist for examining genetic diversity in this species, apart from the small subunit rRNA genes (*11*).

**Figure:**
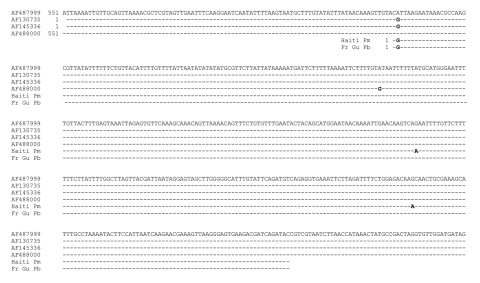
Comparison of Plasmodium malariae (Pm) rRNA gene sequences (GenBank accession nos. AF487999, AF145336, AF488000) and P. brasilianum (Pb) sequences from a monkey from French Guiana (Fr Gu) (AF130735) with isolates from P. malariae of humans from Haiti (Haiti Pm).

## Conclusions

Our results provide conclusive evidence that *P. malariae* is still being transmitted on the island of Hispaniola, which contains the countries of Haiti and the Dominican Republic. *P. malariae* and *P. brasilianum* have been reported in both human and simian hosts in the continental South American states of Suriname, French Guiana, and Brazil (*8*,*12*,*13*), which are located on the northeastern coast of the continent, facing Hispaniola. Although a recent introduction of *P. malariae*, due to movement of persons, is possible, *P. malariae* has likely been present but unreported for a long period because it has been incorrectly diagnosed as *P. vivax*, as has occurred in Suriname (*12*). Indeed, Garnham (*14*) described the ring forms of *P. malariae* in blood films as “rather like those of *P. vivax,*” although less amoeboid and with a more dense rim of cytoplasm.

The movement of *P. malariae* to Jamaica has implications for surveillance throughout the Caribbean and the southeastern United States. *P. malariae* infections can persist without symptoms for long periods, and thus the absence of recent travel history to a malaria-endemic area is not a reliable criterion for ruling out a diagnosis of malaria caused by this species. Our study shows that molecular diagnostic methods can provide the sensitivity and discriminating power required to identify *P. malariae* in such infections.

Furthermore, Jamaica is especially at risk for reintroduction of malaria (as illustrated by the Great Exuma outbreak) because the population is immunologically naive and the competent vector, *Anopheles albimanus,* is endemic there. A reassessment of the risk for *P. malariae* infection in Haiti should be undertaken before control and elimination programs are implemented.
